# GenCLiP: a software program for clustering gene lists by literature profiling and constructing gene co-occurrence networks related to custom keywords

**DOI:** 10.1186/1471-2105-9-308

**Published:** 2008-07-13

**Authors:** Zhong-Xi Huang, Hui-Yong Tian, Zhen-Fu Hu, Yi-Bo Zhou, Jin Zhao, Kai-Tai Yao

**Affiliations:** 1 Cancer Institute, Southern Medical University, Guangzhou, 510515, PR China; 2 Experiment Center, School of Biomedical Engineering, Southern Medical University, Guangzhou, 510515, PR China; 3 Department of Plastic Surgery, Nanfang Hospital, Southern Medical University, Guangzhou, 510515, PR China; 4 Cancer Research Institute, Central South University, Changsha, 410078, PR China

## Abstract

**Background:**

Biomedical researchers often want to explore pathogenesis and pathways regulated by abnormally expressed genes, such as those identified by microarray analyses. Literature mining is an important way to assist in this task. Many literature mining tools are now available. However, few of them allows the user to make manual adjustments to zero in on what he/she wants to know in particular.

**Results:**

We present our software program, GenCLiP (Gene Cluster with Literature Profiles), which is based on the methods presented by Chaussabel and Sher (*Genome Biol *2002, 3(10):RESEARCH0055) that search gene lists to identify functional clusters of genes based on up-to-date literature profiling. Four features were added to this previously described method: the ability to 1) manually curate keywords extracted from the literature, 2) search genes and gene co-occurrence networks related to custom keywords, 3) compare analyzed gene results with negative and positive controls generated by GenCLiP, and 4) calculate probabilities that the resulting genes and gene networks are randomly related. In this paper, we show with a set of differentially expressed genes between keloids and normal control, how implementation of functions in GenCLiP successfully identified keywords related to the pathogenesis of keloids and unknown gene pathways involved in the pathogenesis of keloids.

**Conclusion:**

With regard to the identification of disease-susceptibility genes, GenCLiP allows one to quickly acquire a primary pathogenesis profile and identify pathways involving abnormally expressed genes not previously associated with the disease.

## Background

Biomedical researchers often want to explore pathogenesis and pathways regulated by abnormally expressed genes, such as those identified by microarray analyses. Literature mining is an important way to assist in this task [1]. Many literature mining tools or methods have been developed [2-14] that can extract keywords or gene networks from the literature to functionally group genes and visualize their relationships. However, to our knowledge none of these tools are capable of automatically and easily constructing gene networks among the analyzed genes based on specified keywords, an aspect that would be helpful for investigating disease-associated signaling pathways. Here we report our software program, GenCLiP (Gene Cluster with Literature Profiles), which is based on the two methods provided by Chaussabel and Sher [2] and Jenssen et al. [3]. GenCLiP can cluster functionally related genes based on up-to-date literature profiling and identify gene networks based on specified keywords.

The method of Chaussabel and Sher [2] can be used to analyze gene lists in order to cluster the genes based on up-to-date literature associations. This method first defines terms that occur frequently and exclusively in at least two analyzed genes' up-to-date related literature as keywords, then clusters the analyzed genes based on keyword occurrences. We have added a new feature to this method that allows the user to manually curate keywords through a series of operations including adding/removing, term weighting, defining synonyms, or defining singular/plural forms. Thus, the processed keywords will be more related to the pathogenesis of the specified disease.

The method of Jenssen et al. [3] shows how gene networks can be constructed based on simple co-occurrence. We have added the capacity to limit the extraction of analyzed gene co-occurrences from the literature to only those found in literature that contain certain keywords. This enables the user to readily identify pathways that are specifically associated with the pathogenesis of a particular disease by selecting suitable keywords.

To verify the literature profiling cluster result and the networks related to specified keywords, we have added two features to GenCLiP. One is that GenCLiP can generate two groups of control genes: 1) by using the full gene set to generate a group of negative control genes randomly, and 2) by using the full gene set and the curated keywords to search the database (e.g. PubMed [15] or Entrez Gene [16]) for all related genes to generate a group of positive control genes randomly. The other feature is that GenCLiP can use PubMed search results of the full gene set to perform 10,000 or more random simulations to calculate the probability that a set of randomly picked genes contain the same or more number of related genes (or gene pairs) as the analyzed genes do.

## Implementation

### GenCLiP workflow

**Figure 1:**
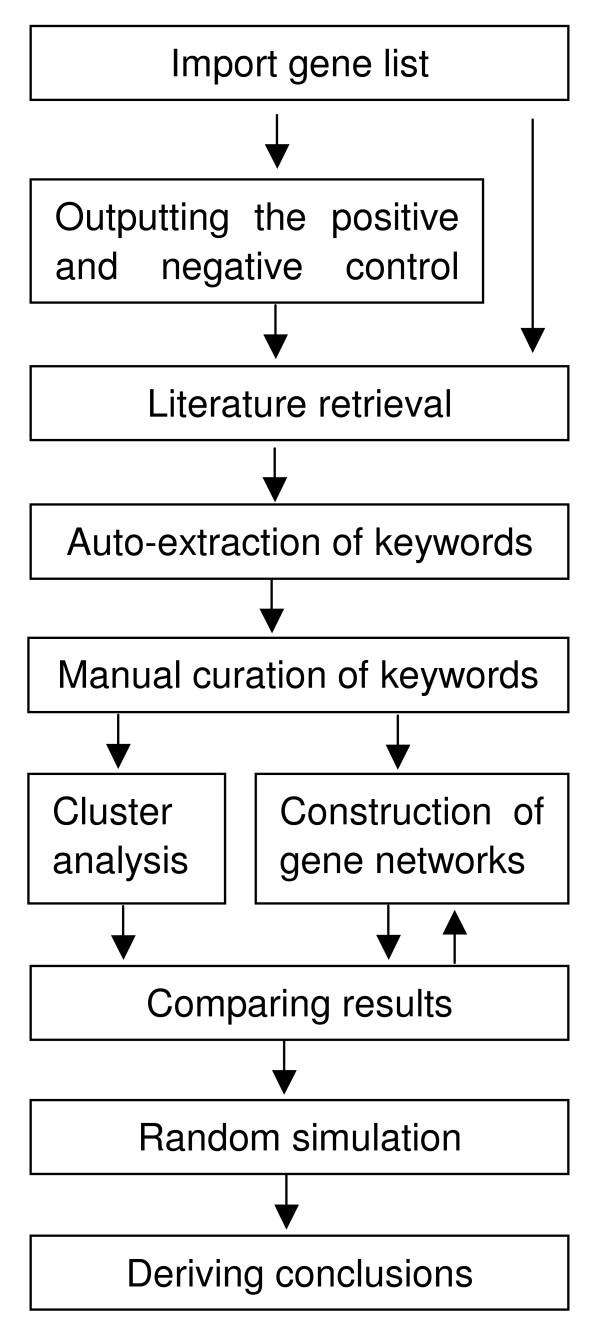
**Workflow of GenCLiP.** The pipeline for the analysis and visualization of the gene list in the context of their underlying functional groups and co-occurrence networks with GenCLiP. See Implementation section (GenCLiP workflow) for a detailed description.

The workflow of GenCLiP is shown in Figure 1. First, a group of positive control genes and a group of negative control genes are generated based on the imported gene list. Then the literature pertaining to each gene of the three groups of genes is retrieved from PubMed. After that, the keywords related to each group of genes are auto-extracted from the literature. The keywords can be manually curated. Then, each group of genes is clustered with the keywords, and the gene co-occurrence networks can be constructed among each group of genes based on certain keywords. After that, the cluster results and the gene networks should be compared among the three groups of genes, and the user can select the keywords that are more related to the positive control genes and the analyzed genes compared to the negative control genes to construct gene networks. Once the analyzed genes and the positive control genes are found to contain more genes (or more complex gene networks) related to certain keywords than the negative control genes, 10,000 or more random simulations are done to decide whether it occurs randomly. Thus, an inference can be obtained for further experiment verification.

### Generation of controls

To generate the negative control genes, the full gene set from which the analyzed genes are derived is used to generate a group of genes randomly. To generate the positive control genes, the full gene set and certain keywords are first used to search the database (e.g. PubMed or Entrez Gene) for all known genes related to the specified keywords. Then, the known-related gene set is used to generate a group of genes randomly. The analyzed genes, the positive control genes, and the negative control genes should have the same number of genes. And the average number of literature per gene for each of them should be comparative. We arbitrarily set that the average number of literature per gene for each group of randomly picked genes should be between 75 and 125% of that for the analyzed genes or as close as possible to that for the analyzed genes. A file recorded the literature number for each of all human genes has been generated and is updated at approximately 6-month intervals. This file is used to calculate the average number of literature per gene for each of the three groups of genes.

### Literature retrieval

To retrieve literature pertaining to each gene of the three groups of genes, the NCBI EUtilities [17], ESearch, and EFetch web services, are used to access the PubMed database [15] for the description [6]. The user can decide whether to provide the gene symbol directly, or provide the gene ID (HUGO [18], Entrez [16], or Unigene [19]). If an alternative gene ID is available, it can be converted to the appropriate input form using MatchMiner [20,21]. Each gene's literature is saved in a text file with the gene's official symbol as the file name. To solve the ambiguity of gene names [22,23], including synonyms (different names for the same gene) and homonyms (different genes or unrelated concepts with the same name), a human gene thesaurus that collected all the aliases for each gene name from the HUGO Nomenclature Committee database [18] and the Entrez Gene database [16] is used. The specificity of each gene name in the thesaurus has been improved by several methods for the description [2,3,5,6]. The thesaurus is discussed in detail in additional files [see Additional file 1].

### Auto-extraction of keywords

Auto-extraction of keywords is performed for the description [2]. Briefly, terms are first extracted from literature titles and abstracts, and their occurrences (number of literature containing a given term divided by the number of total literature) for each gene are calculated. The terms are then filtered systematically using several criteria. (i) Terms with a baseline occurrence (the average occurrence of a set of 250 randomly picked genes, which has been proven unbiased [2]) of more than 5% are eliminated. (ii) Term-occurrence values for each gene are compared to the baseline. The difference cut-off between gene term occurrence and baseline occurrence is set as: cut-off = *t *+ (*k*/*n*) (where *n *is the number of abstracts for a given gene, *t *and *k *are constants, with defaults *t *= 15% and *k *= 1.5). It is noted that if one sets a lower value of t and k, then more terms will pass the filter, but the noise will increase [2]. (iii) Only the terms that pass through the filter for at least two of the analyzed genes are retained. These retained terms are considered keywords.

### Manual curation of keywords

Keywords can be manually curated. The user can remove unrelated keywords and add relevant keywords (single terms or phrases). The user sets the weight for certain keywords that are perceived more important than others. The user defines certain keywords as one synonym entity. The user also determines which keywords have singular/plural forms.

### Clustering analysis

Clustering analysis is performed for the description [2]. Briefly, occurrences of all keywords for each gene pass through the following processes: (i) The occurrence of each keyword in its singular and plural forms is averaged into one unique occurrence; (ii) Each occurrence is multiplied by its weight; (iii) Occurrences of synonyms are averaged into one unique occurrence, and each synonym entity is represented by a keyword. An array file is then generated and used to do clustering analysis with the average linkage hierarchical clustering algorithm for the description [24]. This file can also be used for clustering analysis with publicly available software, such as Cluster 3.0 [25] and SpotFire (Göteborg, Sweden).

### Network construction

Gene co-occurrences are searched from the literature that contains certain keywords. The Neato program in the WinGraphviz software [26] is used to create a two-dimensional layout.

### Random simulation

Random simulation is performed with two steps. First, each gene of the full gene set is used to search PubMed for whether its literature mentions certain keywords, and the resulting PubMed IDs are recorded. Second, for each simulation, the same number of genes as the number of the analyzed genes are randomly picked from the full gene set, and the number of genes (and then gene pairs, i.e. two genes sharing the same PubMed ID) related to the specified keywords are counted. The average number of literature per gene for the randomly picked genes should be comparative with that for the analyzed genes (for details, see the "Generation of controls" section). After 10,000 or more random simulations, if the distribution of the number of related genes (or gene pairs) is similar to the expected normal distribution and the probability that a set of randomly picked genes contain the same or more number of related genes (or gene pairs) as the analyzed genes do is less than 0.05 (i.e. P < 0.05), then it can be inferred that the gene relatedness is not random. [27,28].

### Literature display

The literature containing certain genes and keywords can be searched and displayed with the genes and the keywords coded by different colors.

## Results

GenCLiP was used to analyze a list of 247 differentially expressed genes [see Additional file 2] between keloids and normal skin derived from a sample gene-expression dataset generated by a microarray (CSC-GE, Shenzhen Chipscreen Biosciences Ltd., China) [29]. A keloid is a type of scar that results in an overgrowth of tissue at the site of a healed skin injury [29].

The list of 247 genes were transformed into 234 unique official symbols (13 genes were removed because 10 did not have an official symbol and the other 3 were repeated genes). Two hundred thirty-two of the 234 genes had related literature with an average of 848 literature per gene. The full gene set [see Additional file 3] of the microarray from which the analyzed genes had been derived was used to generate a list of 232 genes randomly [see Additional file 4] with an average of 887 literature per gene. And the full gene set of all human genes was used to search PubMed for all known genes related to the term "keloids". A group of 232 positive control genes [see Additional file 5] with an average of 2,451 literature per gene was then generated from the full known keloids-related gene set. The literature pertaining to each gene of the three groups of genes was then retrieved to the local machine with one text file per gene and one file folder per group of genes. After that, the keywords were auto-extracted from the literature with 502, 505, and 591 keywords for the analyzed genes, the positive control genes, and the negative control genes, respectively [see Additional files 6, 7 and 8] (Table 1).

**Table 1:** Comparing the GenCLiP processing results for the analyzed genes, the negative control genes, and the positive control genes.

	**Analyzed**	**Negative**	**Positive**
Number of genes	232	232	232
Literature per gene	848	887	2,451
Auto-extracted keywords	502	591	505
Associations with auto-extracted keywords	13,943	15,625	31,224
Associations with manually curated keywords	27,221	19,659	81,685
Genes related to 'keloid'	25	9	232
Gene pairs co-occurring with 'keloid'	2	0	434
Genes related to 'hypoxia' and 'fibroblast'	31	19	123
Gene pairs co-occurring with 'hypoxia' and 'fibroblast'	20	3	321

The three groups of genes and their keywords were used to do cluster analysis. The cluster results showed that each of the three keyword lists was divided into many sub-groups (data not shown). Some of these sub-groups, such as sub-group containing keywords "fibroblast" or "collagen", are directly related to the biology of keloids. However, most of these sub-groups, such as sub-groups containing keywords '15', 'kda', and 'mg', are obviously un-related to the biology of keloids [see also Additional files 6, 7 and 8]. The cluster results were compared with each other and showed that there were more keywords related to the biology of keloids in the group of analyzed genes and the group of positive control genes than that in the group of negative control genes (data not shown).

The 502 keywords of the analyzed genes were manually curated further. First, 476 of the 502 keywords that did not seem directly related to keloid biology were manually removed and only 26 keywords remained (Table 2). Furthermore, some of the terms such as 'keloid' and 'scar' that are closely related to keloid pathogenesis were not included as keywords due to low occurrence frequency. Sixteen of them were manually added as keywords (Table 2, marked by asterisks), yielding 41 total keywords. Since some keywords such as 'keloid' and 'scar' might be more related to keloid pathogenesis than the other keywords, higher weights were given to them (Table 2). Therefore, after cluster analysis, genes related to them will be easier to group together. For example, to group keloid-related genes, the weight of the keyword 'keloid' was set from low to high until they can be grouped together (Table 2). Many of the 41 keywords are synonyms, such as 'hypoxia' and 'hypoxic', so the 41 keywords were reset as 16 distinct keyword entities (Table 2). Lastly, some of the 41 keywords might appear in the literature in singular and plural forms (e.g., 'keloid' and 'keloids'), and these should be considered as one keyword.

**Table 2:** Processed keywords for exploring keloid pathogenesis.

**Keyword^a^**	**Plural Flag^b^**	**Weight**	**Synonym Flag^c^**	**Keyword**	**Plural Flag**	**Weight**	**Synonym Flag**
*SCAR	1	100	1	ENDOTHELIAL	0	2	9
*KELOID	1	30,000	2	VASCULAR	0	2	9
*DERMIS	0	11	3	HYPOXIA	0	21	10
SKIN	1	10	3	HYPOXIC	0	20	10
*CUTIS	0	3	3	IMMUNE	0	2	11
*CORIUM	0	2	3	IMMUNOGLOBULIN	0	1	11
*DERMA	0	1	3	*IMMUNOSUPPRESSIVE	0	1	11
*HYPODERMIS	0	1	3	*PARANEOPLASTIC	0	1	12
FIBROBLAST	1	11	4	CARCINOMA	1	1	12
*MYOFIBROBLAST	1	10	4	TUMOR	1	1	12
MATRIX	0	10	5	CANCER	1	1	12
COLLAGEN	1	11	6	APOPTOSIS	0	2	13
PROCOLLAGEN	1	10	6	APOPTOTIC	0	1	13
*HYPERTROPHIC	0	11	7	*GROWTH FACTOR	1	1	14
*HYPERPLASIA	1	2	7	CYTOKINE	1	1	14
PROLIFERATION	1	2	7	*INVASION	0	21	15
INFECTION	1	1	8	*INVADE	0	20	15
INFLAMMATION	1	1	8	ADHESION	1	10	15
INFLAMMATORY	0	1	8	DEGRADATION	0	21	16
ANGIOGENESIS	0	11	9	*COLLAGENASE	1	20	16
ANGIOGENIC	0	10	9				

^a^An asterisk indicates a term that was added manually by the user.

^b^The plural flag ID "1" means the keyword can occur in the literature in its singular or plural form.

^c^Keywords having the same synonym flag ID (larger than zero) are considered one entity.

**Figure 2:**
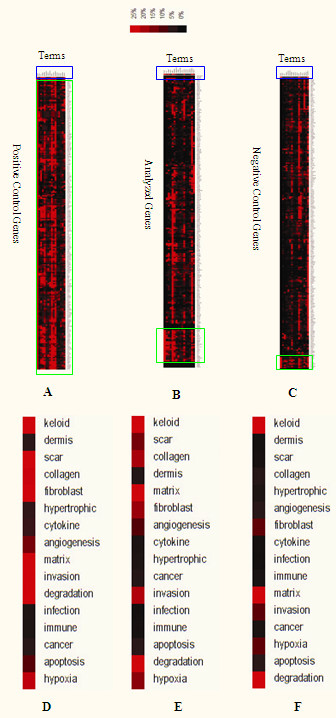
**Literature profiling for three groups of genes.** The clustergram generated for the analysis of patterns of keyword occurrence for the positive control genes (A), the analyzed genes (B), and the negative control genes (C), where blue boxed regions indicate the top bar, and green boxed regions indicate the keloid-related genes. (D-F) Magnification of the blue boxed regions in A, B, and C, respectively, for the positive control genes (D), the analyzed genes (E), and the negative control genes (F), which show the degree of association between keywords and genes.

The 41 curated keywords were used to re-cluster the three groups of genes (Figure 2 and Table 1). The cluster results showed that the positive control genes (Figures 2A, 2D) and the analyzed genes (Figures 2B, 2E) were more highly related to the keywords, whereas the negative control genes (Figures 2C, 2F) were only related to some of the keywords to different degrees. Comparison of the cluster results showed that the keywords 'scar' and 'collagen' were specific to the analyzed genes and the positive control genes, and in a relative sense the keywords 'fibroblast', 'hypoxia', 'angiogenesis', and 'invasion' were more related to the positive control genes and the analyzed genes compared to the negative control genes. These results indicated that the curated keywords and the analyzed genes were indeed related to keloid disease, and the pathogenesis of the analyzed keloid patients might be related to the abnormalities of scars, collagen, fibroblasts, hypoxia, angiogenesis and invasion.

The co-occurrence networks were constructed among each of the three groups of genes based on the curated keyword "keloid" (Table 1). As a result, there were 25 known keloid-related genes reported among the analyzed genes, whereas two pairs of them (*COL14A1 *and *TNC*, *COL1A2 *and *PEPD*) were co-occurred in the literature that mentioned the curated keyword 'keloid' (data not shown). To determine whether the 25 known keloid-related genes were identified randomly, first, each gene of the full gene set of the microarray was used to search PubMed with the curated keyword "keloid". Then, 100,000 random simulations were done. As a result, the distribution of the number of keloid-related genes was found to be similar to the normal distribution, and the probability that a set of 232 randomly picked genes contained more than 24 keloid-related genes was P = 0.00003 (data not shown). Thus, the 232 analyzed genes are related to keloid disease.

**Figure 3:**
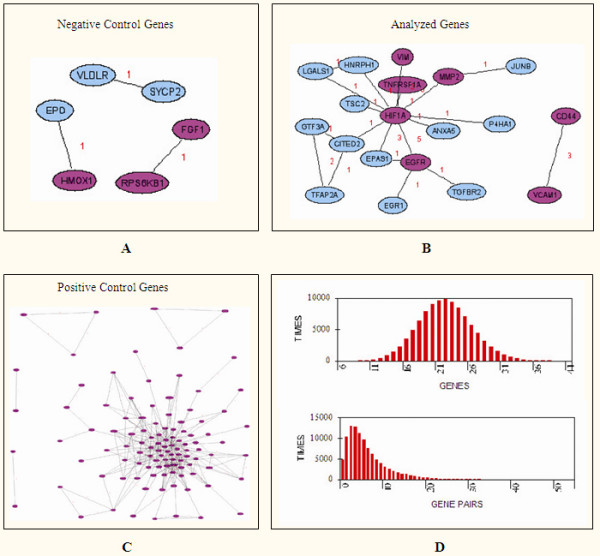
**Searching gene co-occurrence networks using the curated keywords 'hypoxia' and 'fibroblast', and then searching for genes also related to the keyword 'keloid' among the 232 negative control genes (A), the analyzed genes (B), and the positive control genes (C).** The magenta colored node represents known keloid-related gene; the node in sky-blue represents unknown keloid-related gene. (D) Distribution of the number of related gene and gene pairs derived from the random genes were similar to the expected normal distribution.

Exposure of fibroblasts to hypoxia is involved in keloid pathogenesis [29]. Thus, because only a few pairs of genes co-occurred in the literature that mentioned the keyword 'keloid', and because the above clustering analysis showed that the pathogenesis of the analyzed keloid patients might be related to the abnormalities of fibroblasts and hypoxia, gene networks among the three groups of genes were further constructed based on the keywords 'hypoxia' and 'fibroblast' (Table 1 and Figure 3), where genes known-related to the keyword 'keloid' were indicated too. The results showed that there were 31 of the 232 analyzed genes related to the keywords 'hypoxia' and 'fibroblast', while 19 of them (containing 20 pairs of genes) formed gene co-occurrence networks (Figure 3B). The probability that a set of randomly picked genes contain more than 30 genes (or 19 gene pairs) related to the keywords 'hypoxia' and 'fibroblast' was then calculated as P = 0.0228 (or P = 0.01589), and the distribution of the number of related gene and gene pairs was similar to the normal distribution (Figure 3D). Thus, the keywords 'fibroblast' and 'hypoxia' are related to keloid disease and the analyzed genes are engaged in the same networks related to the keywords 'hypoxia' and 'fibroblasts'.

Since the above analysis showed that the two keloid-related genes, *HIF1A *and *MMP2*, co-occurred in the literature that mentioned the keywords 'hypoxia' and 'fibroblast' but not in the literature that mentioned the keyword 'keloid', *HIF1A *and *MMP2 *might be involved in an unknown pathway that is abnormal in keloids. The literature that mentioned the keywords 'hypoxia' and 'fibroblast', and also the genes *HIF1A *and *MMP2 *was thus further searched and displayed. There were three literature obtained (data not shown). Two of the three literature showed that the expression of matrix metallopeptidase 2 (encoded by *MMP2*), which is involved in the breakdown of extracellular matrix in normal physiological processes, is up-regulated by hypoxia through over-expression of hypoxia-inducible factor *HIF1A *in fibroblasts [30,31]. Thus, the pathway (*MMP2 *up-regulated by *HIF1A*) may be involved in the expansion of keloids and merit further experimental study.

## Discussion

Here we show that using GenCLiP, the researcher can gain the pathogenesis profiling of a specified disease and unknown pathways involved in the pathogenesis of the disease from a set of related genes. However, it should be noted that some of the pathways inferred by GenCLiP may already be well established, despite genes never having been mentioned together in any abstract [1]. The user can check them in the curated databases (refer to Pathguide: the pathway resource list [32]) or read the full-text of related papers. Thus, it will be up to the user to decide whether some of the inferred pathways are trivial and thus unworthy of future investigation.

A obvious advantage of GenCLiP over other tools is that all of the associations between the analyzed human genes and the keywords outputted by GenCLiP are up-to-date, because they are calculated de novo. However, the cost is that the processes such as literature retrieval and keywords auto-extraction are time consuming. Generally, the cycle for GenCLiP analysis of a set of experimental data will be several days, especially in developing countries with slow internet speeds. While many of the existing publicly available literature mining tools will provide results to the user immediately. Because they use pre-calculated associations between all human genes and keywords, which are saved in a database that thus allows the user to search the database and obtain the results immediately.

Some of the existing publicly available tools such as PubGene [3] and Ali Baba [7] can also construct gene networks based on keywords. However, the essential differences between GenCLiP and these tools are that: (i) GenCLiP constructs gene networks from the list of analyzed genes, whereas PubGene and Ali Baba construct gene networks from all human genes and only allow the user to query sub-networks containing one or several of the analyzed genes – making the resulting networks quite unrelated to the list of analyzed genes; (ii) for keywords, GenCLiP can use any of the terms or phrases present in abstracts, whereas PubGene and Ali Baba can only use the terms from dictionaries such as MESH, Gene Ontology, and diseases, which may sometimes limit the capability of the user to construct gene networks of interest; and (iii) GenCLiP can use keywords in combination to construct gene networks, whereas PubGene and Ali Baba can only use one keyword to construct a gene network, which limits the capability of constructing gene networks that are highly specific.

In the future, we will develop GenCLiP as a web-based tool. And we will add several features to the next version of GenCLiP, such that: (i) the resulting gene networks will also show the expression value of each node (gene); and (ii) integrating GenCLiP with other data mining tools that can mine pathways from a large set of high-throughput data (such as microarray gene expression data) and the curated pathway databases (such as databases listed in Pathguide [32]) will form a platform [33-36] that can explore the pathogenesis of a specified disease more comprehensively and powerfully.

## Conclusion

In this paper we present an program GenCLiP, a literature mining tool that can cluster a list of genes with keywords that are auto-extracted from their up-to-date related literature and then manually curated by the user. GenCLiP can also generate a group of negative control genes and a group of positive control genes for comparing the cluster results with the analyzed genes to filter out un-specific keywords. GenCLiP allows users to search gene and gene co-occurrence networks related to certain keywords among each of the three groups of genes, and decide whether their associations are random by using a random simulation. Further, GenCLiP can display literature mentioning specified genes and keywords for manual verification of their associations. All these features empower GenCLiP to interpret disease pathogenesis and find novel genes or pathways for further research.

## Availability and requirements

GenCLiP is freely available under the BSD Open Source license for download from  or as additional files to this manuscript [see Additional file 9]. It runs on Windows platform. It is noted that a license is needed to include source code from the GenCLiP Package in commercial software projects.

## Authors' contributions

Z–XH was involved in the development of the method and responsible for development of software as well as for writing the paper. H–YT was involved in the development of method and software. Z–FH was responsible for the generation of microarray data and was involved in the analysis of data. Y–BZ and JZ were involved in the analysis of data and the development of method. K–TY was responsible for the development of the method. All authors read and approved the manuscript.

## Supplementary Material

Additional file 1 Construction of human gene thesaurus. The document contains the detailed discussion of the construction of a human gene thesaurus for literature retrieval. Click here for file

Additional file 2 The 247 analyzed gene list. The data contains an index of the gene abbreviations and full names of the 247 analyzed genes. Click here for file

Additional file 3 The full gene set of microarray. The data contains an index of the gene abbreviations and full names of the full gene set of a microarray (CSC-GE, Shenzhen Chipscreen Biosciences Ltd., China) from which the 247 differentially expressed genes had been derived. Click here for file

Additional file 4 The negative control gene list. The data contains an index of the gene abbreviations of the 232 negative control genes. Click here for file

Additional file 5 The positive control gene list. The data contains an index of the gene abbreviations of the 232 positive control genes. Click here for file

Additional file 6 The auto-extracted keyword list for the 232 analyzed genes. The data contains a group of keywords auto-extracted from the literature pertaining to each of the 232 analyzed genes with default setting. Click here for file

Additional file 7 The auto-extracted keyword list for the positive control genes. The data contains a group of keywords auto-extracted from the literature pertaining to each of the 232 positive control genes with default setting. Click here for file

Additional file 8 The auto-extracted keyword list for the negative control genes. The data contains a group of keywords auto-extracted from the literature pertaining to each of the 232 negative control genes with default setting. Click here for file

Additional file 9 The GenCLiP package. The file is the installation package of GenCLiP. It runs on Windows platform. Click here for file
