# Supplementary material for: GenCLiP: a software program for clustering gene lists by literature profiling and constructing gene co-occurrence networks related to custom keywords
Source: BMC Bioinformatics. 2008 Jul 13;9:308. doi: 10.1186/1471-2105-9-308 (PMC2483997; doi:10.1186/1471-2105-9-308)
Supplement: Additional file 1 — Construction of human gene thesaurus. The document contains the detailed discussion of the construction of a human gene thesaurus for literature retrieval. [file 1471-2105-9-308-S1.doc]

#### Human gene thesaurus

To solve the ambiguity of gene names [1, 2], including synonyms (different names for the same gene) and homonyms (different genes or unrelated concepts with the same name), GenCLiP uses a human gene thesaurus that collected all of aliases for each gene and limited the specificity of each gene with several methods for the description [3-6]. The human gene thesaurus was compiled from the HUGO Nomenclature Committee database [7] and the Entrez Gene database [8]. Human gene symbols (both official and alias), gene names, and product names were included. Gene names were processed as follows. (i) Contents in parentheses were deleted. (ii) Different variant forms of the gene symbols were added by adding/removing a space between the end non-digit and digit character, such as ‘Bcl 2’ versus ‘Bcl2’. (iii) Symbols shorter than three characters (such as ‘CT’, ‘A1’, etc.) were removed. (iv) English word symbols (such as ‘FAT’, etc.) were removed using an English dictionary [4, 5]. (v) Common word gene names (such as ‘protein’, ‘tissue’, etc.) were removed using a baseline occurrence list (provided by D. Chaussabel, personal communication), which has been proven unbiased [6]. If the baseline occurrence of a gene name was greater than 1%, then it was considered common. We chose this somewhat high cutoff percentage because some of the most investigated genes (p53 for example) have a baseline occurrence of 1%. (vi) Common phrase gene names (i.e., those for which each term in the name is a common word, such as ‘novel protein’) with an exceptionally high number of hits (more than 100) were manually curated. (vii) If a gene name was shorter than five characters [3], the same as a cell line name [9], or composed of common words, an assistant (one of the uncommon words or phrases derived from the list of full gene names) was required. It should be noted that some of the above processing steps, such as removal of English words and use of assistant search terms, will reduce sensitivity to some degree. These parameters, however, can be manually corrected in the literature retrieval window of GenCLiP.

#### Evaluation of performance

We have used the above gene thesaurus construction strategy to solve the synonym and homonym problems. To test our gene thesaurus construction strategy, we conducted a PubMed search for 4,999 random human genes using three search strategies for the description [5]: (i) the official symbol for each gene (Symbol), (ii) the official symbol with all its aliases and gene product names (Expanded), and (iii) informative terms only (Filtered). The Expanded search allowed identification of literature information for ~700 additional genes over the number obtained when only the official gene symbols were queried (Table 1). Using the Filtered search terms allowed this addition without adding significantly to the number of queries that returned unreasonable results. In addition to expanding the number of genes that were found in the literature, the Filtered search terms also increased the number of articles found per gene (from an average of 165 articles per gene found by searching with the symbol alone to an average of 363 articles per gene when searching with the filtered terms). These results indicate that our gene thesaurus construction strategy achieved a higher percentage of relevant literature search results for each gene while limiting the addition of irrelevant information.

Table 1. Summary of PubMed hit counts for 4,999 random human genes using different search strategies.

| **Type of primary term^a^** | **Positive results^b^** | **Unreasonable results^c^** | **Articles per gene^d^** |
| --- | --- | --- | --- |
| Symbol | 2,738 | 2 | 165 |
| Expanded | 3,433 | 42 | 1,139 |
| Filtered | 3,353 | 3 | 363 |

^a^The PubMed search was conducted using three search strategies: (i) ‘Symbol’ refers to a search in which each gene was represented by its official symbol; (ii) ‘Expanded’ refers to searches in which each gene was represented by the gene symbol, all its synonyms, and the official gene product name; (iii) ‘Filtered’ refers to searches in which uninformative names were filtered out of the expanded list.

^b^Number of queries that returned at least one result.

^c^Number of queries that returned more than 44,000 results. We used the number 44,000 as a rough estimate of unreasonable results based on the fact that some of the most investigated genes, like p53, appear in less than 44,000 abstracts.

^d^The average number of abstracts per gene—counting only genes that appeared at least once and did not appear in more than 44,000 abstracts.

**References**
